# The protein kinase DYRK1A phosphorylates the splicing factor SF3b1/SAP155 at Thr434, a novel *in vivo *phosphorylation site

**DOI:** 10.1186/1471-2091-7-7

**Published:** 2006-03-02

**Authors:** Katrin de Graaf, Hanna Czajkowska, Sabine Rottmann, Len C Packman, Richard Lilischkis, Bernhard Lüscher, Walter Becker

**Affiliations:** 1 Institute of Pharmacology and Toxicology, Medical Faculty of the RWTH Aachen University, Wendlingweg 2, 52074 Aachen, Germany; 2 Division of Biochemistry and Molecular Biology, Medical Faculty of the RWTH Aachen University, Pauwelstr. 30, 52074 Aachen, Germany; 3 Department of Biochemistry, University of Cambridge, 80 Tennis Court Road, Cambridge CB2 1GA, UK; 4 Genomics Institute of the Novartis Research Foundation, 10675 John J. Hopkins Dr., San Diego, CA 92121, USA

## Abstract

**Background:**

The U2 small nuclear ribonucleoprotein particle (snRNP) component SF3b1/SAP155 is the only spliceosomal protein known to be phosphorylated concomitant with splicing catalysis. DYRK1A is a nuclear protein kinase that has been localized to the splicing factor compartment. Here we describe the identification of DYRK1A as a protein kinase that phosphorylates SF3b1 *in vitro *and in cultivated cells.

**Results:**

Overexpression of DYRK1A caused a markedly increased phosphorylation of SF3b1 in COS-7 cells as assessed by Western blotting with an antibody specific for phosphorylated Thr-Pro dipeptide motifs. Phosphopeptide mapping of metabolically labelled SF3b1 showed that the majority of the *in vivo*-phosphopeptides corresponded to sites also phosphorylated by DYRK1A *in vitro*. Phosphorylation with cyclin E/CDK2, a kinase previously reported to phosphorylate SF3b1, generated a completely different pattern of phosphopeptides. By mass spectrometry and mutational analysis of SF3b1, Thr434 was identified as the major phosphorylation site for DYRK1A. Overexpression of DYRK1A or the related kinase, DYRK1B, resulted in an enhanced phosphorylation of Thr434 in endogenous SF3b1 in COS-7 cells. Downregulation of DYRK1A in HEK293 cells or in HepG2 cells by RNA interference reduced the phosphorylation of Thr434 in SF3b1.

**Conclusion:**

The present data show that the splicing factor SF3b1 is a substrate of the protein kinase DYRK1A and suggest that DYRK1A may be involved in the regulation of pre mRNA-splicing.

## Background

The excision of introns from pre-mRNA is catalysed by the spliceosome, a macromolecular machine consisting of five small nuclear ribonucleoprotein particles (snRNPs) and a large number of non-snRNP proteins [1]. Spliceosome assembly proceeds *via *the step-wise recruitment of U1 snRNP, U2 snRNP, and U4/U6·U5 tri-snRNP on a pre-mRNA as well as multiple rearrangements between the spliceosomal components [1]. After splicing catalysis, the spliceosome dissociates into its snRNP subunits, which take part in ensuing rounds of splicing.

Both spliceosome assembly and splicing catalysis is regulated by reversible protein phosphorylation [1-3]. The best studied targets for phosphorylation are members of the SR family of splicing factors, which contain domains rich in Arg/Ser dipeptides [4]. Several kinases phosphorylate these RS domains and modulate interaction of SR proteins with other proteins during spliceosome assembly [5]. In addition, phosphorylation affects the intranuclear distribution of splicing factors and alternative splice site selection [6-10].

The only non-SR component of the spliceosome known to be phosphorylated during splicing catalysis is SF3b1 (also called SAP155 or SF3b155), one of the subunits of the U2 snRNP-associated complex SF3b [3,11]. SF3b1 is positioned at the spliceosome catalytic center and contacts pre-mRNA on both sides of the branch site [12]. Phosphorylation of SF3b1 appears to be functionally important in the basic splicing reaction as it is detected only in functional spliceosomes and occurs concomitant with splicing catalysis [3]. The N-terminal part of SF3b1 contains abundant Thr-Pro dipeptides motifs which are potential phosphorylation sites of proline-directed kinases like the cyclin-dependent kinases (CDK). Indeed, cyclin E/CDK2 has been shown to phosphorylate SF3b1 *in vitro *and to be associated with the U2 snRNP complex *in vivo *[11].

We have recently identified several splicing factors, including SF3b1, as substrates of the protein kinase DYRK1A [13]. DYRK1A is a nuclear protein kinase that has been localised to the splicing factor compartment [14]. Furthermore, we have previously characterised DYRK1A as a kinase that targets serine/threonine followed by a proline residue [15].

Here we report that DYRK1A efficiently phosphorylates SF3b1 within the TP-rich domain at several sites that are also phosphorylated by endogenous kinases in COS-7 cells. One of these sites, Thr434, was identified as the residue predominantly phosphorylated by DYRK1A *in vitro *and as a major phosphorylation site of SF3b1 *in vivo.*

## Results

### SF3b1 is a high affinity *in vitro *substrate of DYRK1A

**Figure 1:**
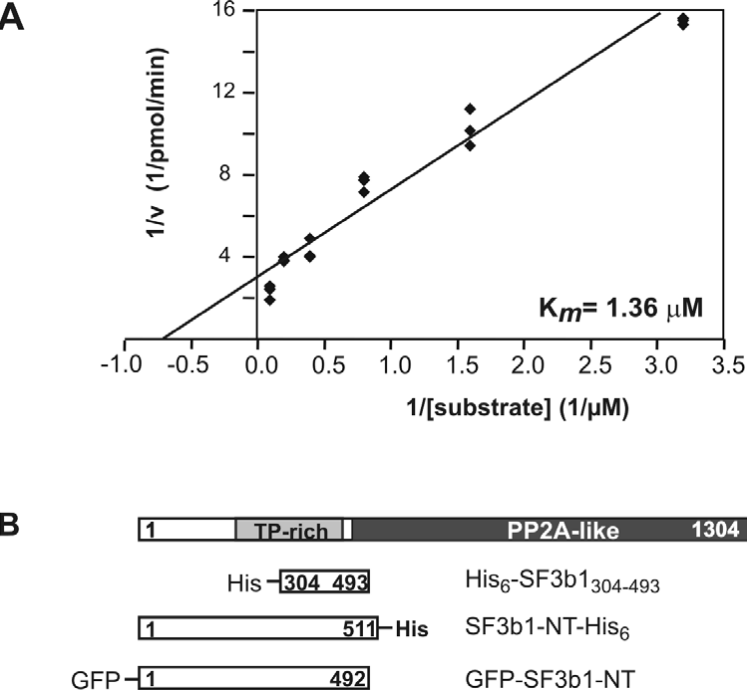
**DYRK1A phosphorylates SF3b1 within the TP-domain**. A, Phosphorylation of His_6_-SF3b1_304–493 _by DYRK1A. – Data from a representative experiment were evaluated by linear regression analysis of the Lineweaver-Burke plot. B, Schematic representation of human SF3b1 and the recombinant fusion proteins used in this study. The carboxyterminal part of SF3b1 consists of 22 nonidentical repeats related to the regulatory subunit A of protein phosphatase 2A (PP2A-like) [3]. Numbers indicate amino acids. TP-rich, region rich in Thr-Pro dipeptides; His, hexahistidine tag.

We have recently identified SF3b1 as an *in vitro *substrate of DYRK1A by screening of a cDNA expression library from human fetal brain [13]. In order to further characterise SF3b1 as a substrate of DYRK1A, we performed a kinetic analysis of the phosphorylation of His_6_-SF3b1_304–493_, the fusion protein produced from the library clone, by GST-DYRK1A-ΔC. The C-terminally deleted mutant of GST-DYRK1A was used for *in vitro*-kinase assays since this construct exhibits the same substrate specificity but is more active than wild type GST-DYRK1A [15,16]. The *K*_*m *_value obtained for total phosphate incorporation into the substrate was 2.16 +/- 1.72 μM (mean of three independent experiments +/- S.E.M.), characterising SF3b1 as a high affinity substrate of DYRK1A. A representative experiment is shown below in Fig. 1A. Notably, His_6_-SF3b1_304–493 _contains 14 Thr-Pro dipeptide motifs (Fig. 1B) which are potential target sites for DYRK1A.

### Phosphorylation of SF3b1 by DYRK1A in COS-7 cells

**Figure 2:**
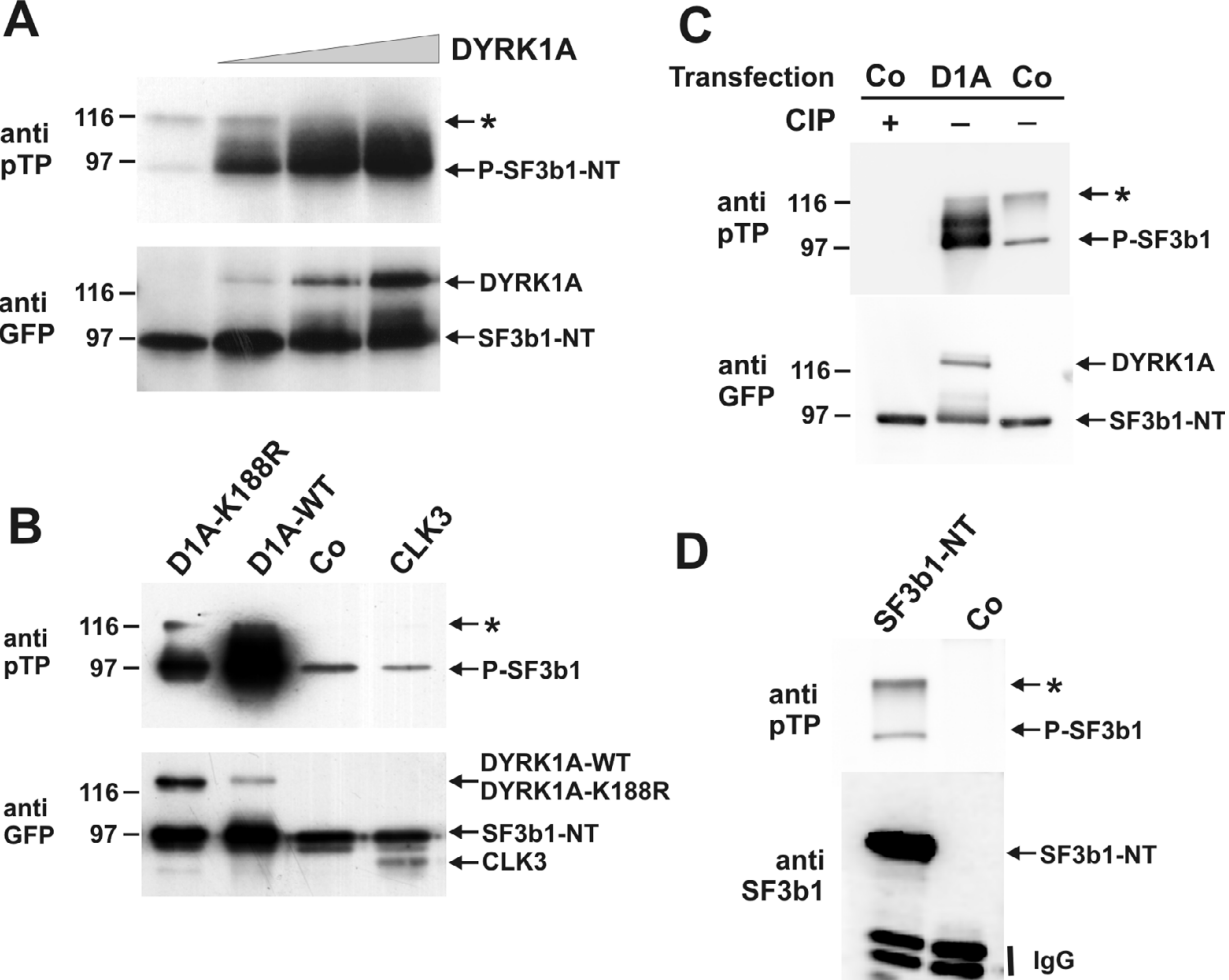
**Phosphorylation of SF3b1 by DYRK1A in COS-7 cells**. COS-7 cells were transiently transfected with expression plasmids for GFP-SF3b1-NT and the protein kinases DYRK1A or CLK3. Two days after transfection, cells were lysed under denaturing conditions and the recombinant proteins were immunoprecipitated with polyclonal GFP antiserum (**A, B, C**) or monoclonal SF3b1 antibody (**D**). Immunoprecipitates were subjected to Western blot analysis with antibodies specific for phosphoThr-Pro (anti pTP), GFP, or SF3b1. **A**, COS-7 cells were transfected with 1 μg of pEGFP-SF3b1-NT and increasing amounts of pEGFP-DYRK1A (0 μg, 0.2 μg, 1 μg and 2 μg of DNA per 6-cm plate). **B**, Cells were transfected with pEGFP-SF3b1-NT and either pEGFP-DYRK1A-K188R, pEGFP-DYRK1A (WT), pEGFP (Co), or pEGFP-CLK3. **C**, Cells were transfected pEGFP-SF3b1-NT and either pEGFP-DYRK1A (DIA) or pEGFP (Co). Cells from one plate were lysed in buffer lacking phosphatase inhibitors, and the lysate was incubated for 1 h at 37°C with 2000 u of calf intestinal phosphatase (CIP) before immunoprecipitation. **D**, Cells were transfected pEGFP-SF3b1-NT (left lane) or were not transfected. Migration of the immunoprecipitating antibody is indicated (IgG). The asterisk marks a slowly migrating form of SF3b1-NT (see text).

In order to assess whether DYRK1A phosphorylates SF3b1 *in vivo *we co-transfected COS-7 cells with expression plasmids for GFP-SF3b1-NT and GFP-DYRK1A. Assuming that DYRK1A may phosphorylate one ore more of the Thr-Pro dipeptides, we took advantage of a commercially available antibody which recognises phosphothreonine C-terminally flanked by proline (pTP) to detect phosphorylation of SF3b1. This antibody detected two bands in the immunoprecipitates from cells overexpressing GFP-SF3b1-NT, of which the lower one (apparent molecular weight of about 95 kD) also reacted with the GFP-specific antibody (Fig. 2A). The difference from the calculated molecular weight (82.4 kD) is possibly due to post-translational modifications. As shown in Fig. 2C, both bands were eliminated by treatment with alkaline phosphatase. Furthermore, the upper band was also found after immunoprecipitation with an SF3b1-specific antibody, but not in untransfected cells (Fig. 2D). Thus, this band most likely represents a highly phosphorylated form of GFP-SF3b1-NT which is present in too low amounts to be detected by the GFP-specific antibody (see also below, Fig. 7B). Co-transfection of DYRK1A caused a very pronounced and dose-dependent increase in the phosphorylation of SF3b1-NT (95kD-band) (Fig. 2A), strongly suggesting that DYRK1A phosphorylates SF3b1 in COS-7 cells. This effect required low amounts of GFP-DYRK1A compared with its substrate GFP-SF3b1-NT, as evidenced by the direct comparison of GFP-immunoreactivity (second lane in Fig. 2A).

To test the specificity of this reaction, we compared the effects of GFP-CLK3 and GFP-DYRK1A on the phosphorylation of SF3b1-NT. Protein kinases of the CLK family are related with the DYRK family and also phosphorylate splicing factors [17]. As a further control, we used GFP-DYRK1A-K188R which carries a point mutation in the ATP binding site and exhibits greatly reduced catalytic activity (1–3% of residual activity [16,18]). As shown in Fig. 2B, co-expression of GFP-CLK3 failed to induce phosphorylation of SF3b1 as compared to GFP alone. As shown by immunodetection with the GFP-specific antibody, GFP-CLK3 was expressed at similar levels as wild type GFP-DYRK1A (see also Fig. 7B). Immunocomplex kinase assays with myelin basic protein as substrate confirmed that GFP-CLK3 was an active protein kinase when expressed in COS-7 cells (data not shown). Unexpectedly, co-expression of DYRK1A-K188R significantly enhanced phosphorylation of SF3b1-NT, although the effect was much weaker than that of the wild type kinase (note also that in the experiment shown DYRK1A-K188R was expressed at a higher level than wild type DYRK1A). The result that a mutant of DYRK1A with reduced activity (K188R), but not the related kinase CLK3, enhanced threonine phosphorylation of SF3b1-NT is evidence of the specificity of this reaction.

### Comparison of the phosphorylation of SF3b1 by DYRK1A and cyclin E/CDK2

**Figure 3:**
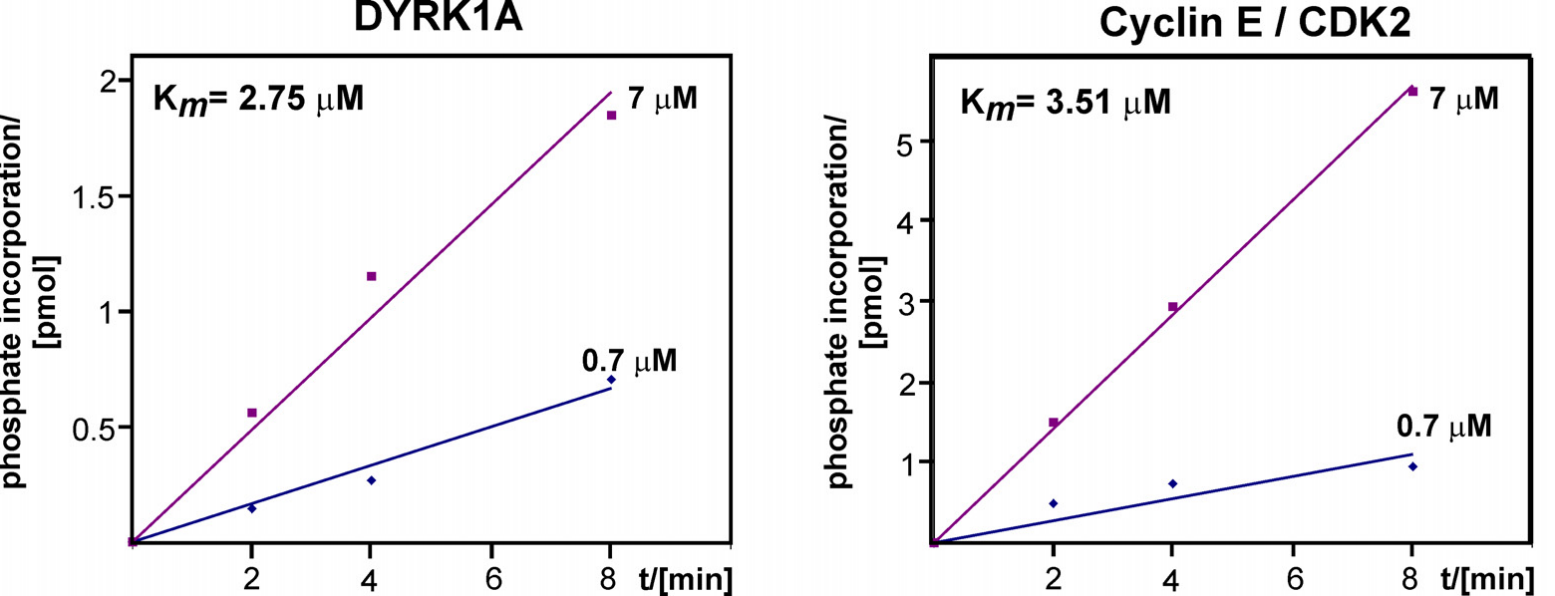
**Kinetic analysis of SF3b1 phosphorylation by DYRK1A and CDK2**. SF3b1-NT-His_6 _was phosphorylated with GST-DYRK1A-ΔC or cyclin E/CDK2 at two different substrate concentrations (0.7 and 7 μM). Phosphate incorporation was measured at different time points (2, 4 and 8 minutes). The slope of the straight line reflects the velocity of phosphorylation at the different substrate concentrations. The calculated K_*m *_values are indicated. The experiment was repeated with similar results.

SF3b1 is phosphorylated concomitant with or just after catalytic step one of the splicing reaction [3]. The kinase responsible for this phosphorylation during splicing catalysis has not been characterised to date, but Seghezzi *et al*. [11] have identified SF3b1 as a potential target of cyclin E/CDK2 complexes. In order to compare the phosphorylation of SF3b1-NT-His_6 _by DYRK1A and cyclin E/CDK2, we performed a preliminary kinetic analysis of both reactions by measuring the velocities of phosphate incorporation at two different substrate concentrations (0.7 and 7 μM). The approximate *K*_*m *_values calculated from the results shown in Fig. 3 are very similar for both kinases (2.75 μM for DYRK1A and 3.51 μM for cyclin E/CDK2) and indicate that both kinases have a high affinity for SF3b1.

**Figure 4:**
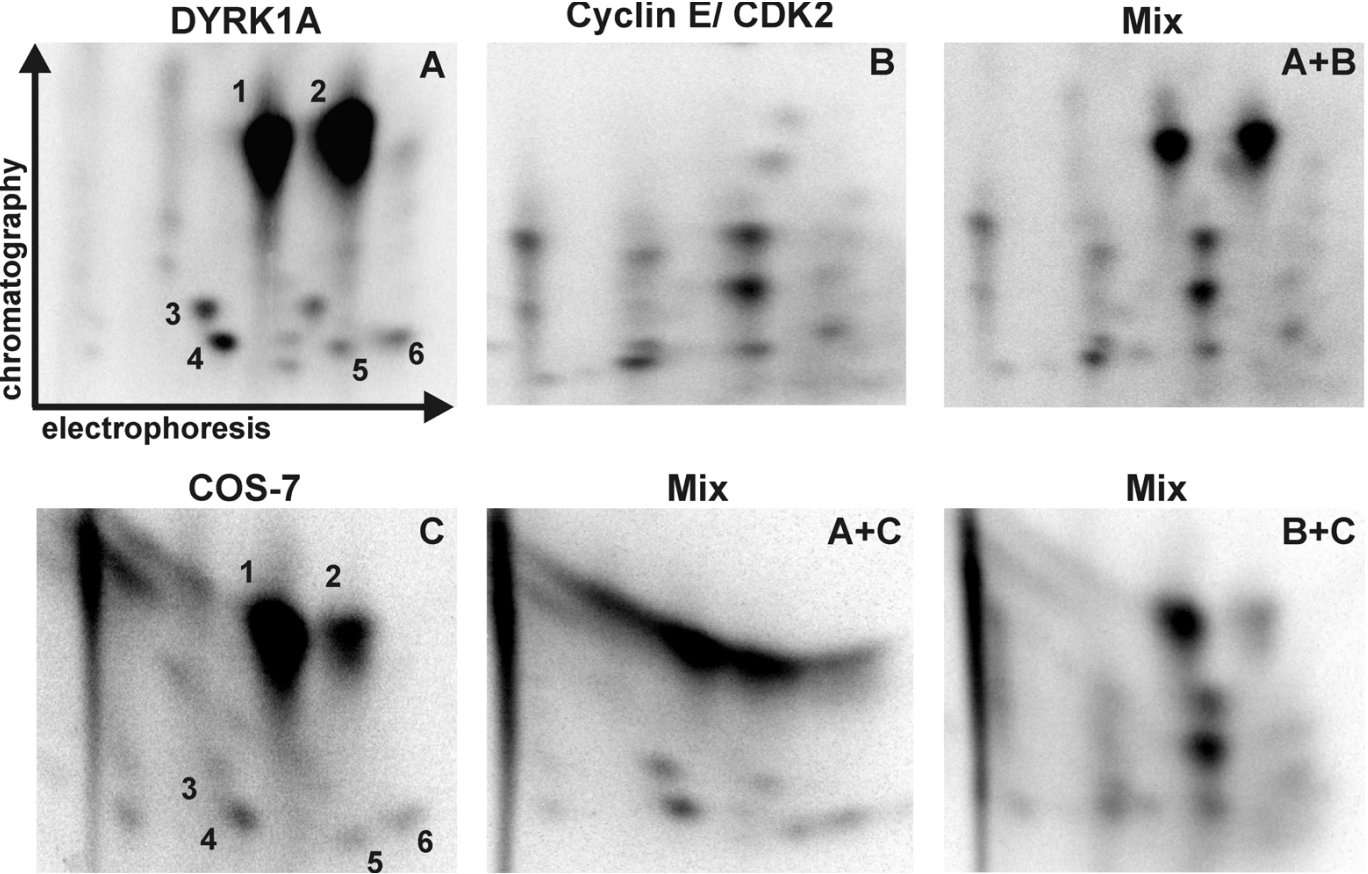
**DYRK1A, but not cyclin E/CDK2 phosphorylates SF3b1 *in vitro *on phosphopeptides comigrating with the endogenous phosphopeptides from COS-7 cells**. SF3b1-NT-His_6 _was labelled *in vitro *by GST-DYRK1A-ΔC (A) or cyclin E/CDK2 (B). GFP-SF3b1-NT was immunoprecipitated from COS-7 cells after metabolic labelling with ^32^PO_4 _(C). Recombinant proteins were subjected to two-dimensional phosphopeptide mapping. To verify that spots detected in different experiments are identical, samples were mixed and analysed on the same plate **(A+B**, **A+C**, **B+C)**. Numbers label those spots that were both present in panels A and C. The panels show only the relevant area of the plates.

To answer the question whether both kinases target the same phosphorylation site(s) in SF3b1, we generated phosphopeptide fingerprints. SF3b1-NT-His_6 _was phosphorylated by either GST-DYRK1A-ΔC or cyclin E/CDK2 *in vitro*, and tryptic peptides of SF3b1-NT were analysed by two-dimensional peptide mapping. The pattern of phosphopeptides derived from DYRK1A-labelled SF3b1 (Fig. 4A) differed completely from the pattern obtained by cyclin E/CDK2 (Fig. 4B), and mixing of the peptides from both experiments revealed no detectable comigration of phosphopeptides (A+B). This result indicates that both kinases phosphorylate different sites in SF3b1.

### DYRK1A phosphorylates SF3b1 *in vitro *on physiologically relevant sites

**Figure 5:**
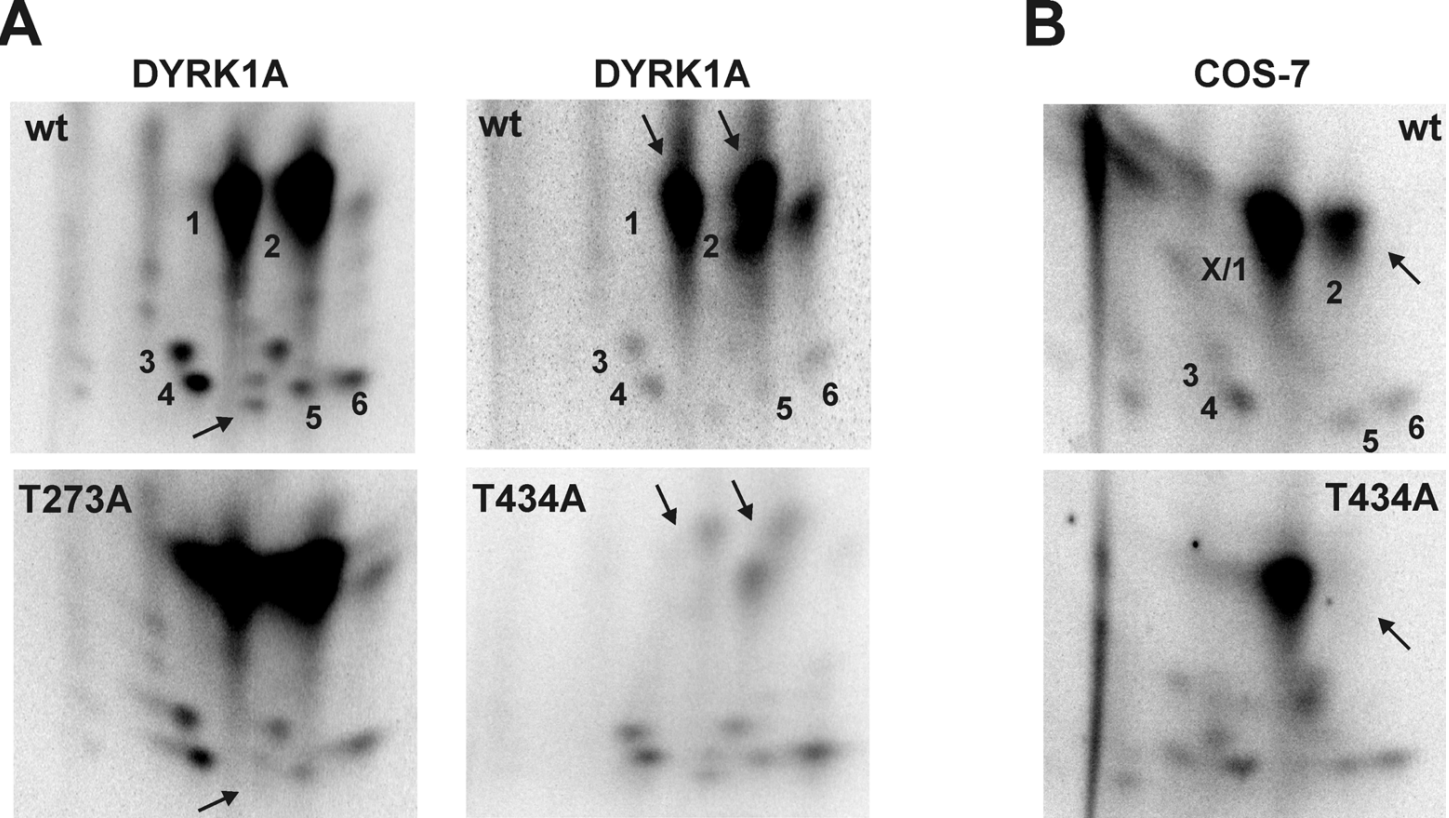
**SF3b1 is phosphorylated at Thr434 by endogenous kinases in COS-7 cells and *in vitro *by DYRK1A**. A, SF3b1-NT-His_6 _(wt) and point mutants of Thr273 (T273A) or Thr434 (T434A) were phosphorylated by GST-DYRK1A-ΔC *in vitro*. B, GFP-SF3b1-NT (wt) or the alanine mutant of Thr434 (T434A) were expressed in COS-7 cells, metabolically labelled with ^32^P and purified by immunoprecipitation with a GFP-specific antiserum. Phosphopeptide maps were generated as described above. Arrows point to phosphopeptides that are absent in the mutants. X marks a spot apparently superimposing spot1 (see text).

Next we asked whether the phosphorylation pattern of SF3b1 *in vivo *better matches the *in vitro*-pattern obtained with DYRK1A or with cyclin E/CDK2. COS-7 cells were transfected with pEGFP-SF3b1-NT and metabolically labelled by incubation with ^32^P-orthophosphate. Phosphopeptide mapping of the immunoprecipitated GFP-SF3b1-NT fusion protein showed that the *in vivo*-phosphorylation pattern (Fig. 4C) strikingly resembled the phosphorylation pattern obtained by *in vitro*-phosphorylation with DYRK1A (Fig. 4A). Six of the spots on the *in vivo*-map matched phosphopeptides generated by DYRK1A *in vitro *and comigrated in the map of a mixed sample (Fig. 4A+C), strongly suggesting that the phosphopeptides generated *in vitro *by DYRK1A are identical with those generated *in vivo*. Unlike *in vitro*, however, spot 1 was much more intense than spot 2. A possible explanation for this difference is a superposition of signals derived from spot 1 and a comigrating phosphopeptide (spot X) that is phosphorylated *in vivo *by a kinase other than DYRK1A (see below, Fig. 5B). No match was detectable between the cyclin E/CDK2 phosphopeptide map and the *in vivo *map (Fig. 4B+C). This result provides evidence that the major part of the phosphorylation within the Thr-Pro-rich domain of SF3b1 is catalysed by DYRK1A or a related kinase with similar substrate specificity. However, it cannot be excluded that relevant CDK2 sites escaped detection because phosphopeptides were lost during purification or were poorly soluble in the running buffers.

### Identification of SF3b1 phosphorylation sites

His_6_-SF3b1_304–493 _was phosphorylated with GST-DYRK1A *in vitro *and tryptic peptides were analysed for phosphorylation by tandem mass spectrometry (MS^2^). Two phosphorylated peptides were identified: (1) VLPPPAGYVPIRTPAR, containing Thr426 (underlined) as the phosphoamino acid; the phosphorylated residue completely inhibited tryptic cleavage at the preceding Arg whereas in an unphosphorylated sample, this cleavage occurred freely. (2) KLTATPTPLGGMTGFHMQTEDR (with both Met residues in the sulphoxide form – a side reaction of preparation). MS^2 ^and MS^3 ^analysis of this peptide indicated that the phosphorylation was confined to either of the first two threonines of the peptide (Thr432 or Thr434) but from the data the labelled residue could not be distinguished. This was because the predicted fragment ions needed to resolve this question laid beyond the dynamic range of the ion-trap instrument. Attempts to examine smaller MS^2 ^ions further by MS^3 ^to gain access to this region were unproductive, as were secondary digest attempts with chymotrypsin. We considered Thr434 the more likely target because DYRK1A is a proline-directed kinase. Therefore we prepared alanine mutants of Thr426 and Thr434 by site directed mutagenesis of SF3b1-NT. In addition, Thr273 and Thr303 were mutated because the surrounding sequences of both threonines (Thr273: G**R**GDT_273_**P**; Thr303: TE**R**DT_303_**P**) matched known target sequences of DYRK1A (**R**XXS/T**P**[15]; **R**XS/T**P**[19]).

The mutant proteins were phosphorylated with GST-DYRK1A-ΔC *in vitro *and analysed by peptide mapping. Mutation of Thr434 resulted in the loss of the two most prominent spots (spots 1 and 2; right panels of Fig 5A), indicating that Thr434 is the major phosphorylation site for DYRK1A. The existence of two different phosphopeptides containing Thr434 can be explained by incomplete tryptic cleavage as the MS analysis showed this peptide to exist with and without the lysine at the N-terminus. Such ragged N- or C-termini can be expected when an XRKX sequence is cleaved by trypsin. The absence of one spot in the phosphopeptide map of the T273A mutant identified Thr273 as one of the minor *in-vitro *phosphorylation sites of DYRK1A (Fig. 5A, arrow in the left panel). The mutants T426A and T303A yielded the same pattern of spots as the wild type protein (data not shown). The failure to detect the VLPPPAGYVPIRTPAR phosphopeptide containing Thr426, which was identified as a phosphorylated residue by MS, may be due to poor solubility of this peptide under the conditions applied.

Next we asked whether Thr273 and Thr434 are *in vivo *phosphorylation sites of SF3b1. The respective point mutants of GFP-SF3b1-NT were metabolically labelled in COS-7 cells and subjected to phosphopeptide mapping. Analysis of SF3b1-NT-T273A did not reveal differences between the wild type and the mutated protein (data not shown). In contrast, one of the major phosphopeptides (spot 2) was absent in the map of GFP-SF3b1-NT-T434A as compared to the wild type protein (Fig. 5B). This result confirms our conclusion that spot 2 represents the same phosphopeptide in the *in vitro *and the *in vivo*-maps (Fig. 4). We assume that the absence of the other peptide (spot 1) is masked by a comigrating phosphopeptide (spot X). Spot X is lacking in SF3b1-NT-T434A after phosphorylation by DYRK1A *in vitro*, hence this phosphopeptide appears to harbour the only major phosphorylation site not recognised by DYRK1A. These data indicate that Thr434 in SF3b1 is phosphorylated by endogenous kinases in COS-7 cells.

### Overexpression of DYRK1A increases phosphorylation of SF3b1 at in vivo-phosphorylation sites

**Figure 6:**
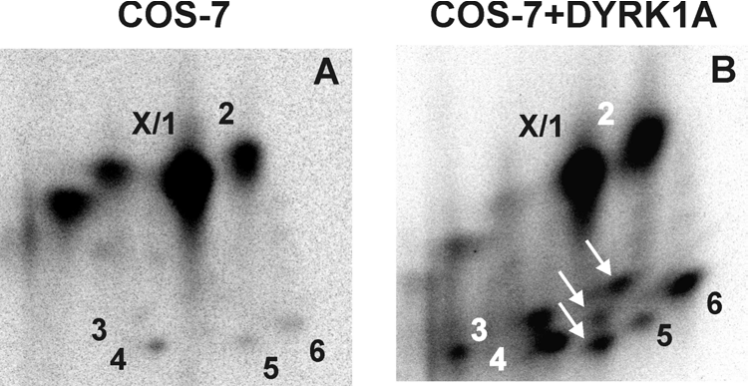
**Overexpression of DYRK1A increases the phosphorylation of endogenous sites in COS-7 cells**. GFP-SF3b1-NT was expressed in COS-7 cells either alone (A) or coexpressed with GFP-DYRK1A (B) and metabolically labelled with ^32^P. SF3b1 fusion proteins were subjected to phosphopeptide mapping as above. Intensities of peptide maps A and B were adjusted to spot X/1.

As shown in Fig. 2A, overexpression of DYRK1A increases the phosphorylation of SF3b1 in COS-7 cells. To investigate whether DYRK1A targets the same sites that are already phosphorylated *in vivo*, we compared the phosphopeptide map of GFP-SF3b1-NT phosphorylated by endogenous kinases in COS-7 cells with the phosphopeptides obtained after cotransfection of GFP-DYRK1A. As shown in Fig. 6, intensities of at least five peptides (spots 2–6) increased upon coexpression of GFP-DYRK1A relative to spot X/1. It should be noted that the comparison with spot X/1, which includes the DYRK1A-phosphorylated spot 1, underestimates the degree of the increase caused by cotransfection of DYRK1A. In addition, three new spots appeared that were not detectable when SF3b1 was labelled without coexpression of DYRK1A (arrows). This result demonstrates that DYRK1A can phosphorylate other residues in addition to Thr434 that are endogenous phosphorylation sites.

**Figure 7:**
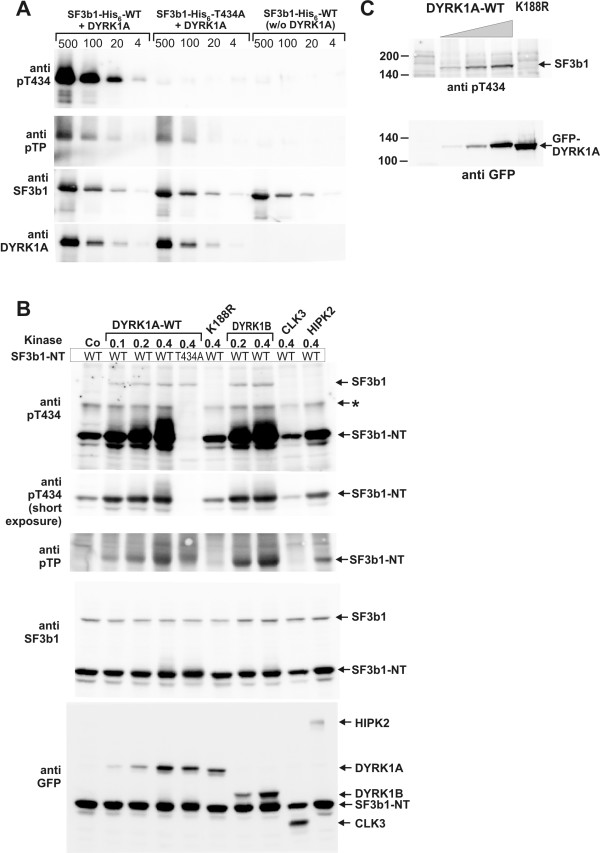
**Detection of Thr434 phosphorylation with the help of a phosphorylation site-specific antibody**. A, Verification of antibody specificity. – Recombinant SF3b1-NT-His_6 _(WT) or the T434A point mutant thereof were phosphorylated *in vitro *by GST-DYRK1A-ΔC or incubated under the same conditions in the absence of GST-DYRK1A-ΔC. The indicated amounts of SF3b1-NT-His_6 _(500 ng, 100 ng, 20 ng, 4 ng) were separated by SDS-PAGE and subjected to Western blot analysis with antibodies specific for pT434, pThrPro (pTP), and SF3b1. B, Phosphorylation of Thr434 *in vivo*. – COS-7 cells seeded in 6-well plates were co-transfected with expression plasmids for wild type GFP-SF3b1-NT (WT) or the T434A mutant (0.6 μg/well) and either GFP (Co) or GFP fusion constructs (0.1 μg, 0.2 μg or 0.4 μg/well) of the indicated protein kinases (wild type DYRK1A or the K188R point mutant, DYRK1B, CLK3, HIPK2). The total amount of transfected DNA was kept constant by addition of vector DNA where appropriate. Two days after transfection, total cellular lysates were prepared and subjected to Western blot analysis with antibodies specific for pT434, pThrPro (pTP), SF3b1, or GFP. A shorter exposure of the top panel is shown below because the most intense signals exceeded the linear range of the detection camera. A slowly migrating form of SF3b1-NT is marked by an asterisk (*). C, Nuclei were purified from COS-7 cells transfected with expression plasmids for wild type GFP-DYRK1A (1 μg, 2 μg or 4 μg/6-cm plate) or the point mutant K188R. Nuclear proteins were subjected to Western blot analysis with the indicated antibodies.

### Phosphorylation of Thr434 in endogenous SF3b1

In order to facilitate detection of phosphorylated Thr434, we raised a polyclonal antiserum against a peptide comprising residues 429–439 of SF3b1, phosphorylated at Thr434. The affinity-purified antibody recognised wild type SF3b1-NT-His_6 _after *in vitro-*phosphorylation by DYRK1A, but not the unphosphorylated protein or SF3b1-NT-His_6_-T434A (Fig. 7A). In contrast, the commercial pThrPro-specific antibody also bound to other phosphorylated ThrPro motifs in the T434A mutant of SF3b1. This result shows that the pT434-directed antibody exhibits high specificity for this phosphorylation site in SF3b1.

The anti-pT434 antibody was then used to study the phosphorylation of Thr434 in transfected COS-7 cells. To assess the specificity of the reaction, several nuclear protein kinases were tested in parallel with DYRK1A for their capacity to enhance phosphorylation of Thr434 in GFP-SF3b1-NT. DYRK1B is the kinase most closely related to DYRK1A (85% of identical amino acids in the catalytic domain [20]). HIPK2 (homeodomain-interacting protein kinase 2) was selected as a more distant member of the DYRK family (42% identity) [21], and CLK3 is a kinase known to phosphorylate splicing factors (see above). As shown in Fig. 7B, the pThr434-specific antibody detected SF3b1-NT in cells that did not overexpress DYRK1A (leftmost lane). This result is consistent with the labelling of spots 1 and 2 by endogenous kinases in COS-7 cells (Fig. 4C, 5B, and 6A). Signal intensity was dose-dependently enhanced by co-expression of DYRK1A or DYRK1B but not DYRK1A-K188R. SF3b1-T434A (lane 5) was not recognised by the antibody, confirming that the antibody was indeed specific for phosphoThr434. Notably, co-expression of HIPK2, but not CLK3, also resulted in an increased phosphorylation of Thr434 in SF3b1-NT. A second band (marked by an asterisk in Fig. 7B) could also be identified as a form of SF3b1-NT because of its absence in cells transfected with SF3b1-NT-T434A. As noted above (Fig. 2A), it is likely that this band represents a posttranslationally modified form of the protein.

In addition to the recombinant SF3b1-NT protein, the anti-pT434 antibody labelled a band with an apparent molecular mass of 150 kDa that was only detectable in lysates of cells overexpressing catalytically active DYRK1A or DYRK1B. This band co-migrated with the endogenous SF3b1 protein as identified by a commercially available antibody. As shown in Fig. 7C, the 150-kDa band was also detected in a nuclear protein fraction purified from DYRK1A-overexpressing COS-7, further supporting the identification as SF3b1. These data provide evidence that DYRK1A and DYRK1B can phosphorylate the full length, endogenous SF3b1 protein in intact cells. In contrast, overexpression of HIPK2 did not enhance phosphorylation of Thr434 in SF3b1, suggesting that this kinase cannot phosphorylate the endogenous protein in the spliceosome.

### Phosphorylation of SF3b1 by endogenous DYRK1A

**Figure 8:**
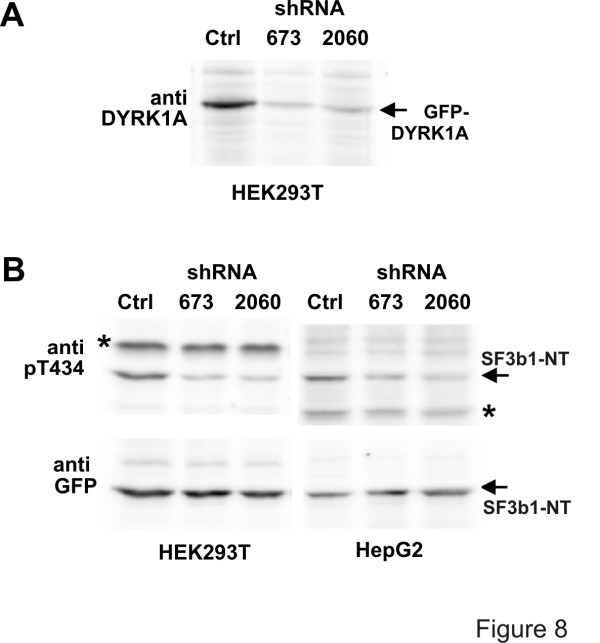
**Downregulation of endogenous DYRK1A reduces phosphorylation of Thr434 in SF3b1**. A, Test of shRNA vectors for DYRK1A knockdown. – HEK293T cells seeded in 6-well plates were co-transfected with expression plasmids for GFP-DYRK1A (0.2 μg/well) and either empty vector (Ctrl) or plasmids expressing to different small hairpin RNAs directed against DYRK1A (673 or 2060; 0.8 μg DNA/well). Two days after transfection, nuclear extracts were prepared and subjected to Western blot analysis with a DYRK1A-specific antibody. B, Effect of DYRK1A knockdown on Thr434 phosphorylation. – HEK293T cells or HepG2 cells were co-transfected with the expression plasmid for GFP-SF3b1-NT (0.5 μg/well) and the pSUPER constructs (0.8 μg/well). Total cellular lysates were subjected to Western blot analysis with antibodies specific for pThr434 or GFP. The asterisks mark unspecific bands (*).

In order to assess the role of endogenous DYRK1A in the phosphorylation of SF3b1, we constructed two plasmids expressing small hairpin RNA (shRNA) for specific downregulation of human DYRK1A. The target sequences were carefully selected to avoid potential effects on DYRK1B mRNA. As shown in Fig. 8A, transient transfection of either one of the shRNA constructs efficiently reduced the level of GFP-DYRK1A, suggesting that they should also downregulate endogenous DYRK1A which is expressed at much lower levels. Next we determined the effect of the shRNA constructs on the phosphorylation of Thr434 in SF3b1-NT in two different human cell lines (Fig. 8B). Transient transfection of either construct resulted in a marked reduction of Thr434 phosphorylation, indicating that DYRK1A is the major Thr434 kinase in HEK193T cells and in HepG2 cells.

## Discussion

The splicing factor Sf3b is an integral part of U2 snRNP and plays an essential role during spliceosome assembly and recognition of the intron's branch point. One of the components of SF3b, SF3b1, is known to be reversibly phosphorylated during splicing catalysis [3], suggesting that protein kinases play a role in the regulation of splicing. Previous studies have shown that cyclin E/CDK2 complexes associate with spliceosomal proteins *in vivo*, and that CDK2 phosphorylates SF3b1 *in vitro *[12,22]. Here we provide evidence that the protein kinase DYRK1A phosphorylates SF3b1 *in vitro *and *in vivo*.

The N-terminal part of Sf3b1 harbours a large number of Thr/Pro dipeptide motifs within a 240-amino acid region preceding the carboxyterminal repeat domain (Fig. 1). Both DYRK1A and CDK2 are proline-directed kinases, *i.e. *they phosphorylate serine or threonine residues followed by a proline residue [15,23]. It has been shown that cyclin E/CDK2 phosphorylates SF3b1 *in vitro *at multiple sites within the TP-rich domain [22]. Here we demonstrate that SF3b1 is phosphorylated by DYRK1A and cyclin E/CDK2 *in vitro *with similarly high affinity, but at different sites. Strikingly, the majority of the *in vivo*-phosphorylation sites within the N-terminal domain of SF3b1 corresponded to sites phosphorylated by DYRK1A *in vitro*, and overexpression of DYRK1A also enhanced the labelling of these phosphopeptides *in vivo*. Importantly, overexpression of DYRK1A resulted in the increased phosphorylation of Thr434 in endogenous SF3b1, indicating that enzyme and substrate come into contact in living cells. However, in contrast to cyclin E/CDK2, DYRK1A does not appear to be stably associated with SF3b1 as we failed to detect the interaction in pulldown assays (data not shown).

Our conclusion that DYRK1A is the major SF3B1 kinase in asynchronously growing COS-7 cells is not in contradiction to previous reports that CDK2 phosphorylates SF3b1 [11,22] but rather complements these studies. Seghezzi *et al. *reported that only about 30% of the SF3b1-phosphorylating activity in immunoprecipitated SF3b1 complexes was inhibited by the CDK inhibitor p21, leading the authors to suggest "the existence of other kinases in the (...) complex" [11]. Boudrez *et al*. [22] also observed that SF3b1 kinase activity in lysates from COS-1 cells was only partially suppressed by the CDK inhibitor roscovitine. It is well possible that the relative contribution of different kinases to phosphorylation of SF3b1 varies in different experimental systems (nuclear extracts, cellular extracts, immunoprecipitated splicing complexes). In our *in vivo*-phosphopeptide maps, no spot corresponded to a site also phosphorylated by cyclin E/CDK2 *in vitro*, indicating that CDK2 did not contribute detectably to phosphate incorporation into SF3b1 under these conditions. In contrast, 6 phosphopeptides matched spots that were obtained after phosphorylation with DYRK1A *in vitro*, indicating that DYRK1A or another protein kinase with similar substrate specificity, *e.g. *DYRK1B [15,20], catalyses the phosphorylation of SF3b1 in COS-7 cells at multiple sites. Overexpression of DYRK1B caused indeed an increase in the phosphorylation of Thr434 in SF3b1. Other members of the DYRK family, DYRK2 and DYRK3, are primarily localised in the cytosol [24] and are thus unlikely to phosphorylate SF3b1. The proposed role of DYRK1A as a regulator of an essential splicing factor is in agreement with its ubiquitous expression [24], the evolutionary conservation of DYRK kinases throughout the eukaryotic kingdoms, and the embryonic lethality of mice homozygous for a targeted deletion of the *Dyrk1a *gene [25]. In contrast, DYRK1B has a more restricted pattern of expression, and DYRK1B deficient mice are viable (S. Leder, M. Moser and W. Becker, unpublished data). Further studies will be necessary to reveal the roles of DYRK1A and DYRK1B in splicing.

Our experiments do not formally exclude the possibility that another nuclear kinase produces the phosphorylation pattern observed in COS-7 cells. We have tested HIPK2 as a DYRK-related kinase and found that this kinase was indeed able to phosphorylate Thr434 in GFP-SF3b1-NT. However, overexpression of HIPK2 did not cause phosphorylation of Thr434 in endogenous SF3b1, making it unlikely that this kinase targets SF3b1 *in vivo*. Moreover, downregulation of DYRK1A by RNA interference led to a marked reduction of Thr434 phosphorylation in HEK293T and in HepG2 cells, providing evidence that in these cell lines DYRK1A is the major kinase that targets this phosphorylation site. It should be noted that one predominant *in vivo*-phosphopeptide (spot X in Fig. 5B) was neither labelled by DYRK1A nor by CDK2 *in vitro*, providing evidence that at least one more kinase phosphorylates SF3b1.

The present data identify Thr434 as the major phosphorylation site of DYRK1A in SF3b1. This site does not exactly match the consensus recognition site for DYRK1A as previously determined in peptide assays [15,26] since no arginine is present at position -2 or -3 relative to the phosphorylation site. However, *in vitro*-assays with a peptide mimicking the sequence context of Thr434 showed that this target sequence is similarly well recognised as a peptide designed according to the consensus phosphorylation sequence for DYRK1A (K. de Graaf, R. Frank and W. Becker, unpublished data).

At present we can only speculate on the effects of the phosphorylation of SF3b1. Protein-protein interactions of SF3b1 with U2AF35/65, the SF3b component p14, and nuclear inhibitor of protein phosphatase 1 (NIPP1) have been mapped to the TP-rich domain [12,22,27]. NIPP1 contains a phosphothreonine-binding forkhead-associated (FHA) domain, and the binding to NIPP1 has been shown to depend on the phosphorylation of SF3b1 by cyclin/CDK complexes [22]. Coexpression of DYRK1A failed to alter the binding of SF3b1-NT to NIPP1 in pulldown assays (data not shown), most likely because CDKs and DYRK1A phosphorylate different sites within SF3b1. It should also be noted that Thr434 is located at the C-terminal end of the TP-rich domain and was absent in some of the constructs to which protein interactions had been mapped in the studies mentioned above. The location of Thr434 in the hinge region between the TP-rich domain and the C-terminal domain makes it tempting to speculate that phosphorylation of this residue may regulate the conformational changes of SF3b that have been proposed to be required for binding of the RNA [28].

## Conclusion

The present data indicate that DYRK1A and/or DYRK1B phosphorylate specific threonine residues within the TP-rich domain of the spliceosomal protein SF3b1. Phosphorylation of SF3b1 has previously been shown to be increased during splicing catalysis [3] and in mitosis [22]. Further work will be necessary to reveal the role of DYRK-related kinases under these conditions and which effects they may have on the function of the spliceosome.

## Methods

### Antibodies

The following antibodies were commercially obtained: rabbit polyclonal antibody for GFP (Molecular Probes, Eugene, USA) and monoclonal antibodies for GFP and SF3b1/SAP155 (MBL, Nagoya, Japan) and phosphothreonine-proline (p-Thr-Pro-101; Cell Signaling Technology, Beverly, MA, USA). The p-Thr-Pro-101 antibody reacts with proteins phosphorylated on the Thr-Pro motif in an otherwise highly context-independent fashion (characterisation by the supplier). Horseradish peroxidase-coupled secondary antibodies were purchased from Perbio Science, Bonn, Germany (anti rabbit IgG, anti mouse IgG/IgM) and Amersham Bioscience (anti mouse IgG). A rabbit polyclonal antibody specific for SF3b1 phosphorylated at Thr434 was raised against the peptide RKLTApTPTPLG (where pT indicates phosphothreonine). The antiserum was purified by affinity chromatography on CNBr-activated Sepharose to which the phosphopeptide antigen had been attached covalently and passed down a CNBr-Sepharose column to which the corresponding unphosphorylated peptide had been coupled (custom immunisation and antibody purification by BioGenes, Berlin, Germany). The purified antibody was used for immunodetection on Western blots at a dilution of 1:200 (0.5 μg/ml).

### Expression plasmids and isolation of recombinant proteins

The plasmids for bacterial expression of GST-DYRK1A-ΔC and GST-DYRK1A-cat and the mammalian expression clones for GFP-DYRK1A, GFP-DYRK1A-K188R, GFP-DYRK1B-p69 and GFP-CLK3 have been described earlier [13,18,20,24]. The plasmid encoding GFP-HIPK2 was kindly provided by M.L. Schmitz (Bern, Switzerland) [29]. An expression plasmid for a His_6_-tagged fragment of SF3b1 (pQE-SF3b1_304–493_, encoding amino acids 304 to 493; numbering according to O75533 was previously isolated from a human fetal brain expression library in a screen for substrates of DYRK1A [13]. A construct (pET28a-SF3b1-NT) coding for amino acids 1 to 511 of human SF3b1 fused to a C-terminal His_6_-tag was generated by PCR cloning (vector pET28a, Novagen, Madison, WI, USA). pEGFP-SF3b1-NT expresses the amino acids 1 to 492 of human SF3b1 in the pEGFP-C1 vector system (Clontech, Palo Alto, CA, USA). Catalytically active human cyclin E/CDK2 complexes E were expressed in insect cells and purified as described [30].

GST- and His_6_-tagged fusion proteins were expressed in *E. coli *and affinity purified using glutathione S-Sepharose 4B (Amersham Bioscience) or nickel-charged nitrilotriacetic acid agarose beads (Qiagen, Hilden, Germany). Proteins were eluted under native conditions (reduced glutathione or imidazol). His_6_-tagged proteins were purified by gel filtration through a Sephadex G25 column (NAP™-5 column, Amersham Bioscience) and equilibrated in 10 mM Tris pH 7.4, 100 mM NaCl. Point mutants of pET28a-SF3b1-NT were produced with the help of QuikChange™ Site-Directed Mutagenesis Kit (Stratagene, La Jolla, CA) and verified by sequencing. Point mutants of pEGFP-SF3b1-NT were made by subcloning of the mutated cDNAs into pEGFP-SF3b1-NT.

### RNA interference

pSuper vectors [31] were constructed that direct the synthesis of small interfering RNA specific for two different 19-bp target sequences within the human DYRK1A mRNA (bp673–691, GCACAGATAGAAGTGCGAC and bp 2060–2079, CGACTTCTTCCTCGACATC, numbering refers to EMBL:U52373).

### Kinetic analysis of SF3b1 phosphorylation

The *K*_*m *_value of the phosphorylation of His_6_-SF3b1_304–493 _by GST-DYRK1A-ΔC was determined as detailed previously [15]. Apparent *K*_*m *_values of three independent experiments each done at five different substrate concentrations were derived by (nonlinear) fitting of the data into the Michaelis-Menten equation with the help of the GraphPad Prism 1.03 program (GraphPad Software, San Diego, CA, USA). R^2 ^values for the non-linear fitting were always greater than 0.9. Only for the graphical representation in Fig. 1A data were evaluated by linear regression. For a comparative kinetic analysis, SF3b1-NT-His_6 _was phosphorylated by either GST-DYRK1A-ΔC (1 unit/ml) or cyclin E/CDK2 (400 units/ml) in the presence of 50 μM ATP (66.6 μCi/ml) and phosphate incorporation was measured at the time points indicated in Fig. 3. The velocities (v_1 _and v_2_) of phosphate incorporation by both kinases were determined at substrate concentrations of S_1 _= 0.7 μM and S_2 _= 7 μM, and approximate *K*_*m *_values were calculated using the transformed Michaelis-Menten equation *K*_*m *_= (v_2 _– v_1_)/((v_1_/[S_1_]) – (v_2_/[S_2_])). One unit of DYRK1A is that amount which catalysed the phosphorylation of 1 nmol of the synthetic peptide DYRKtide (at 100 μM) in 1 min at 30°C [13]. One unit of cyclin E/CDK2 is the amount of catalytically active kinase complex that incorporates 1 pmol phosphate in 5 μg histone H1 in 30 min at 30 °C in CDK2 kinase buffer (50 mM HEPES pH 7.5, 10 mM MgCl_2_, 1 mM sodium vanadate, 10 mM NaF).

### Cell culture, cell lysates, immunoprecipitations and immunoblotting

HEK293T and COS-7 cells were grown in Dulbecco's modified Eagle's medium (DMEM) high glucose, supplemented with 10 % fetal calf serum (FCS). Phosphate-free DMEM was obtained from Sigma-Aldrich (catalogue number D3656) and supplemented with 3.7 g/l NaHCO_3_, 0.11 g/l sodium pyruvate and 10 % dialyzed phosphate free FCS (Sigma-Aldrich, catalogue number F0392). The cells were transfected using FuGENE (Roche, Mannheim, Germany) as suggested by the manufacturer. For the detection of phosphorylated proteins, cell lysis and immunoprecipitation were done under denaturing conditions as described earlier [13]. For analysis of nuclear proteins (Figs. 7C and 8A), cells on a 6-cm plate were lysed by incubation in 1 ml of 20 mM Hepes pH 7.4, 150 mM NaCl, 1.5 mM MgCl_2_, 0.02% NP40 for 10 min on ice. Nuclei were collected by low speed centrifugation (1.300 × *g*, 1 min), washed in the lysis buffer, and nuclear proteins were prepared for gel electrophoresis by incubation in SDS sample buffer at 96°C.

### Mass spectrometry

Proteins were separated by SDS gel electrophoresis and stained with Coomassie Blue. Cut bands were digested with trypsin and the resulting peptides analysed by electrospray mass spectrometry on a ThermoFinnigan LCQ Classic ion-trap instrument using static nanospray delivery, as previously described [16]. Additional MALDI analyses were performed on a Waters TofSpec 2E instrument using alpha-cyano-4-hydroxycinammic acid matrix.

### Two-dimensional phosphopeptide mapping

About 4 μg of SF3b1-NT-His_6 _or its mutant versions were phosphorylated *in vitro *by GST-DYRK1A-ΔC (1.5 units/ml) or CDK2/cyclin E (500 units/ml) in the respective kinase buffers supplemented with 10 μM [γ-^32^P]ATP (100 μCi/ml) and 100 mM NaCl. For metabolic labelling of GFP-SF3b1-NT, transfected COS-7 cells (in 6 cm-diameter plates) were incubated with 200–400 μCi/plate of carrier-free H_3 _^32^PO_4 _(Hartmann Analytic GmbH, Braunschweig, Germany) as described previously [13]. After incubation for 2.5 h, SDS lysates were prepared and the GFP-tagged proteins were immunoprecipitated with the help of a polyclonal anti-GFP antiserum [13]. The *in vitro*-phosphorylated SF3b1 and the immunoprecipitates containing *in vivo*-phosphorylated SF3b1 were purified by SDS-PAGE. The labelled proteins were recovered from cut gel slices, digested with trypsin and subjected to two-dimensional phosphopeptide mapping as detailed previously [32]. Thin-layer electrophoresis on cellulose plates (first dimension) and subsequent thin-layer chromatography were run in acidic pH-1.9 buffer (2.2% formic acid/7.75% acetic acid).

## List of Abbreviations

The abbreviations used are: CDK, cyclin-dependent kinase; GFP, green fluorescent protein; GST, glutathione S-transferase; pTP, phosphothreonine-proline, shRNA, small hairpin RNA

## Authors' contributions

KdG carried out most of the experiments and drafted the manuscript. HC performed the experiments shown in Figs. 7 and 8. SR devised conditions for the phosphopeptide mapping. LCP performed the mass spectrometry analysis. RL prepared cyclin E/CDK and established the assay conditions. BL participated in the design of the fingerprinting experiments and final editing of the manuscript. WB conceived of and planned this study and edited the manuscript. All authors read and approved the final manuscript.
